# Investigation of the Existence of Supplier-Induced Demand in use of Gastrostomy Among Older Adults: A Retrospective Cohort Study

**DOI:** 10.1097/MD.0000000000002519

**Published:** 2016-02-08

**Authors:** Toshiki Maeda, Akira Babazono, Takumi Nishi, Midori Yasui, Yumi Harano

**Affiliations:** From the Department of Healthcare Administration and Management, Graduate School of Healthcare Sciences, Kyushu University, Japan.

## Abstract

The aim of this study is to clarify whether there is small area variation in the use of gastrostomy that is explained by hospital physician density, so as to detect the existence of supplier-induced demand (SID).

The study design is a retrospective cohort using claim data of Fukuoka Late Elders’ Health Insurance, submitted from 2010 to 2013. Study participants included 51,785 older adults who had been diagnosed with eating difficulties. We designated use of gastrostomy as an event. Multilevel logistic analyses were then used to investigate the existence of SID.

After controlling for patient factors, we found significant regional level variance in gastrectomy use (median odds ratio [MOR]: 1.72, 1.37–2.51). Hospital physician density was significantly positively related with gastrostomy (adjusted OR of hospital physician density: 1.75, 1.25–2.45; *P *< 0.001). MORs were largely reduced for the input variable of hospital physician density.

We found that the small area variation in use of gastrostomy among older adults could be explained by hospital physician density, which might indicate the existence of SID.

## INTRODUCTION

After peaking in 2008, the Japanese population as a whole has been decreasing; however, the older population has been consistently increasing. The proportion of adults >65 years old in Japan is now up to approximately one-fourth of the total population, resulting in what is known as a “super-aging” society.^1^ Cerebrovascular accident (CVA), dementia, malignancies, and infections such as pneumonia are prevalent among older adults, as a result of cumulative molecular and cellular damage.^2^ These conditions consequently induce difficulty with intake of adequate nutrition. Gastrostomy for feeding has been commonly used among this population.^3–5^ The All Japan Hospital Association reported there were an estimated 260,000 older adults in Japan with a surgically implanted feeding tube.^6^ However, several studies have found that gastrostomy for older patients, particularly those with advanced dementia, was not associated with prevention of aspiration,^7–9^ recovery from pressure ulcers,^7,10^ or lower mortality.^7,9,11,12^ The Japan Geriatrics Society recently issued a guideline recommending gastrostomy for older adults with eating difficulties only after consideration of whether it would contribute to an improved prognosis or quality of life.^13^ However, it has been reported that the proportion of older patients who were evaluated for swallowing ability before feeding tube placement was as low as 22.9%,^14^ which suggests that pre-evaluation and indications for gastrostomy might not be appropriate. It is well known that gastrostomy feeding tube insertion was relatively profitable in Japan before revision of the national fee schedule in 2014, approximately 1000 USD (100 JPY = 1 USD) per procedure.^15^ Furthermore, inconsistent information about and explanation of the need for gastrostomy is common among medical staff and physicians, and often leads to insufficient understanding on the part of families or caregivers when deciding if the procedure is necessary.^14,16,17^ This situation could influence gastrostomy use even more than patient characteristics, in addition to economic incentives known as supplier-induced demand (SID).

There are various tools for detecting the existence of SID. They include analysis of small area variation and physician density.^18^ We can infer the existence of SID if small area variation can be found, which could in turn be explained by physician density, independent of patient characteristics. Thus, the aim of this study was to clarify whether there is small area variation in gastrostomy use that can be explained by physician density, so as to detect the existence of SID.

## MATERIALS AND METHODS

### Study Design and Participants

The design of this study was a retrospective cohort. In Japan, those aged ≥75 years, or those aged 65 to 74 years with a specific disability, are eligible for the Late Elders’ Health Insurance. We used claim data submitted to Fukuoka Late Elders’ Health Insurance from 2010 to 2013. First, we identified 51,785 participants who had been diagnosed as having difficulty with eating, excluding rule-out diagnoses. We defined placement of a gastrostomy feeding tube as an event that occurred after diagnosis. We categorized eating difficulties according to the International Disease Classification 10th revision (ICD-10), as follows—F50: Eating disorders; J69: Pneumonitis due to solids and liquids; R13: Dysphagia; R630: Anorexia; and R633: Feeding difficulties. We defined a surgically implanted gastrostomy feeding tube as procedure code 150170550 or 50171610. We excluded patients who had a gastrostomy procedure before being diagnosed with any type of eating difficulty. Patients with the following malignancies were also excluded—C00-C14: malignant neoplasms of the lip, oral cavity, and pharynx; C15: malignant neoplasm of the esophagus; and C16: malignant neoplasm of the stomach.

This study was approved by the Institutional Review Board of Kyushu University (Clinical Bioethics Committee of the Graduate School of Healthcare Sciences, Kyushu University).

### Definition of Variables

We identified sex, age, neurological comorbidities, other comorbidities, year of diagnosis, and economic status as patient factors. We established three age categories: <80 years, 80 to 89 years, and ≥90 years. We defined neurological comorbidities as CVA, dementia, extrapyramidal and movement disorders, and other neurological diseases. We applied the Charlson comorbidity index (CCI)^19^ to define CVA and dementia. Extrapyramidal and movement disorders were defined according to the ICD-10, as G20-G26: extrapyramidal and movement disorders. Other neurological disorders were defined as follows—G10-G13: systemic atrophies primarily affecting the central nervous system; G35-G37: demyelinating diseases of the central nervous system; and G70-G73: diseases of the myoneural junction and muscle. We further defined malignancy as other comorbidity using the CCI, except for those malignancies mentioned above. Other than for CVA, dementia, and malignancy, the possible CCI scores were 0, 1 to 2, or 3 or more. Year diagnosed was categorized as fiscal year (FY) 2009 or earlier, FY2010, FY2011, FY2012, or FY2013. Although the data used in this study were submitted between 2010 and 2013, diagnoses before this data submission period retained their diagnosis year and month; hence, we created a variable FY2009 or earlier. With regard to economic status, participants were categorized into low, middle, and high levels according to prior year incomes.^20^ We combined middle and high economic levels into a category of middle–high status because the proportion of patients at high economic levels was extremely low.

We used physician density^21^ by secondary tier of medical care (STM) as regional factors. STM is the unit of secondary care governed by a prefecture according to Japan's Medical Service Law. Each prefecture must set its own STMs; Fukuoka Prefecture has 13 STMs. Gastrostomy is mainly performed in a hospital setting, and hospital physician density reflects health care resources related to gastrostomy. Therefore, in this study, we defined physician density as hospital physician density. Furthermore, greater hospital density indicates more acute care resources. Hospital physician density was transformed into a binary variable. We used the number of hospital physicians according to the Hospital Report of e-Stat (2011), which was created by the Ministry of Internal Affairs and Communications. Population data were obtained from 2010 census data.

### Statistical Analysis

We calculated the percentage of gastrostomy procedures performed by sex, age, neurological comorbidities, other comorbidities, year of diagnosis, economic status, and hospital physician density. We then quantified the strength of relationships using odds ratios (ORs). We performed multilevel logistic analyses with patient factors as level 1 and regional factors as level 2. We first constructed a null model. We then developed model 1, which included patient factors such as sex, age, neurological comorbidities, other comorbidities, diagnosis year, and economic status. Subsequently, we constructed model 2 that included patient factors and hospital physician density. The Akaike information criterion (AIC) was used for goodness of model fit. We used an intraclass correlation coefficient (ICC) for similarity within groups and the median odds ratio (MOR) for variance between groups, in addition to regional level variance. The ICC is the ratio between STM variance and total variance; interpretation of the ICC in a logistic model is limited.^22^ The MOR is defined as the median of a set of ORs obtained when comparing 2 randomly chosen regions with different random effects.^23^ The MOR can be interpreted as the median increased risk of moving from a region with lower risk to one with higher risk, assuming the same patient covariates.^23^ If the MOR is equal to 1, there are no differences between regions for the probability of gastrostomy.^24^ If there were strong regional level differences, the MOR would be larger and the regions involved would be relevant for area variations in the individual probability of gastrostomy.^24^ MOR is computed on the typical OR scale and is therefore comparable to OR for other patient-level factors.^24^

The equation for MOR is: 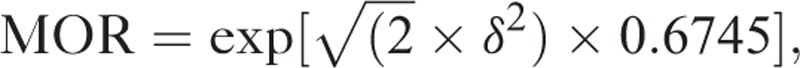


where  
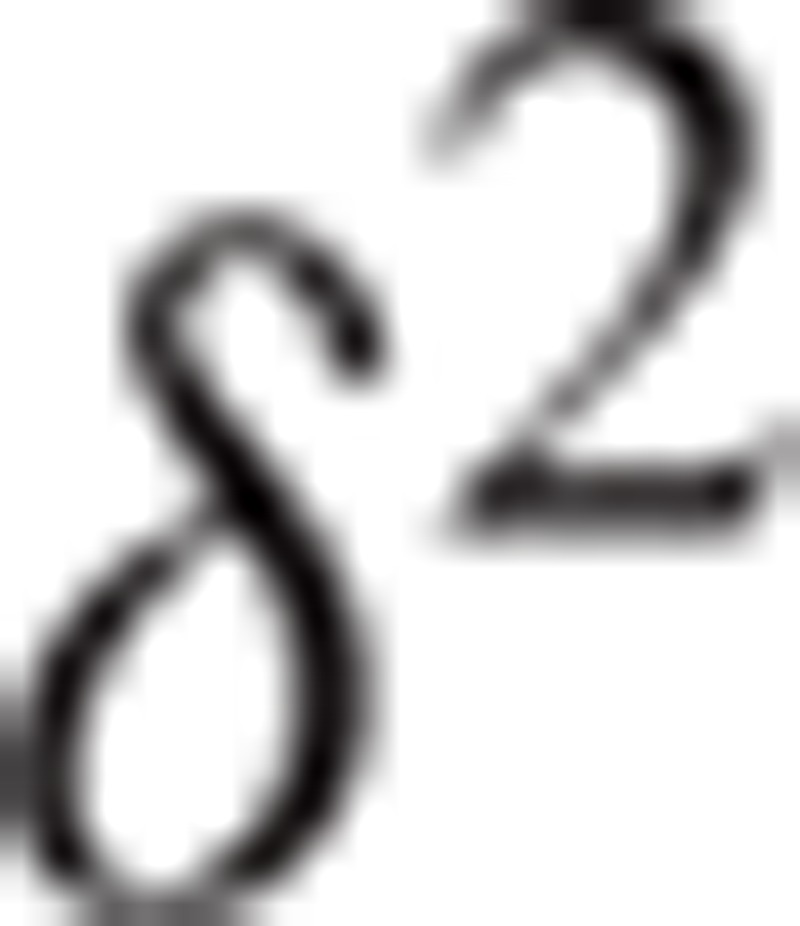
 is the regional level variance.^25^

All reported *P* values were 2-tailed, and the level of significance was set at *P* < 0.05.

We used Stata statistical software, Release 14 (StataCorp, College Station, TX) for the statistical analyses.

### Sensitivity Analysis

For sensitivity analysis, we constructed model 3 by replacing tertile age in model 2 with quantile age (<|80, 80≦<|85, 85≦<|90, 90≦), model 4 by replacing tertile age in model 2 with continuous age, and model 5 by replacing binary economic status in model 2 with tertiles that were very low, low, and middle–high.

## RESULTS

### Regional Information

Information of the study population, number of gastrostomy procedures performed, and number of hospital physicians per 100,000 (hospital physician density) by STM are shown in Table 1. STMs 1 and 12 had markedly more patients with gastrostomy than the other STMs. The number of study participants in STMs 1 and 12 were 11,358 and 13,382, respectively. STMs with the most gastrostomy procedures were STMs 1 and 13, which were 2.4-fold greater than that with the fewest procedures (STM 5). STMs with the highest hospital physician density were STM 6 (331.3), STM 9 (237.0), STM 1 (223.8), and STM 12 (213.8). The ratio of the highest hospital physician density to the lowest was approximately 3.9-fold.

**TABLE 1:**
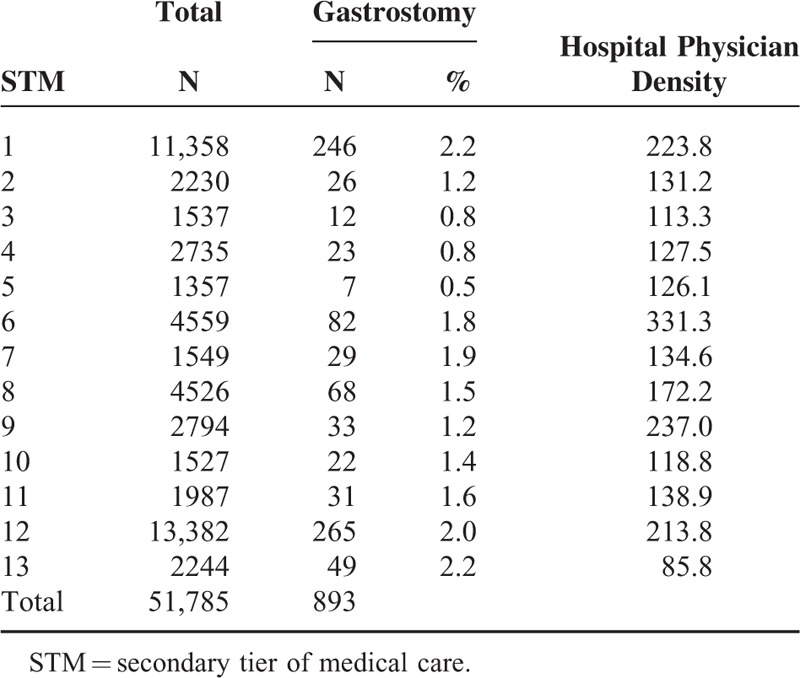
Study Population, Number of Gastrostomy Procedures, and Hospital Physician Density, by Secondary Tier of Medical Care

### Results of Patient Factors, Regional Factors, and Crude ORs for Gastrostomy Use

Table 2 shows sex, age, neurological comorbidities, other comorbidities, year of diagnosis, economic status, and hospital physician density by gastrostomy and crude OR for use of gastrostomy. The number and percentage of gastrostomy procedures were 893 and 1.7% of the entire population (n = 51,785). The proportion of gastrostomy among female patients was significantly lower than that among males. Patients in the 80 to 89-year age group had significantly more gastrostomy procedures than the other age groups, whereas those ≥90 years had significantly fewer procedures than patients aged <80 years. Those with neurological comorbidities had a significantly higher proportion of gastrostomy. Conversely, patients with malignancies had a significantly lower proportion of gastrostomy. Economic status was not significantly related to use of gastrostomy. With respect to regional factors, higher hospital physician density was significantly associated with gastrostomy (number of hospital physicians: OR 1.48, 1.25–1.76; *P* < 0.001).

**TABLE 2:**
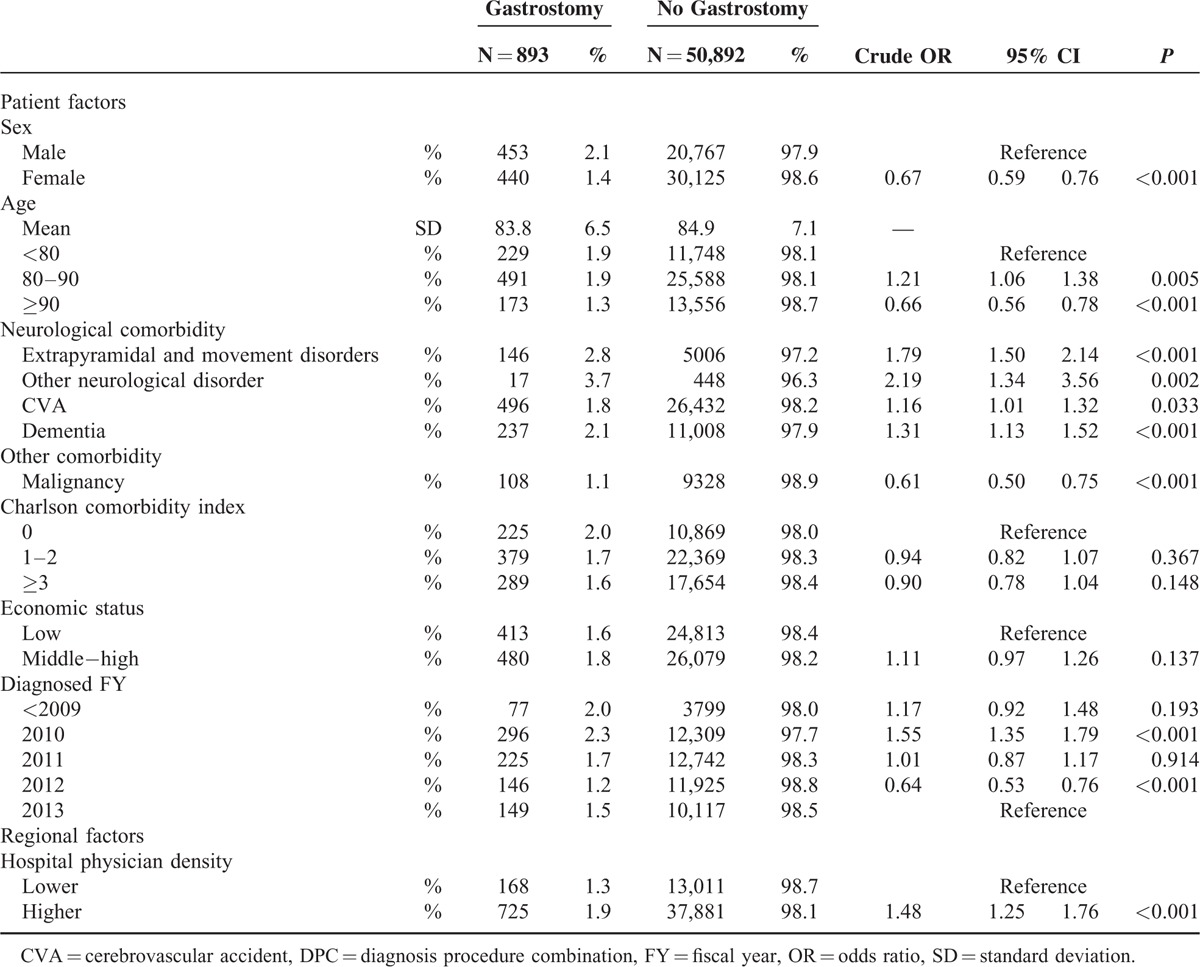
Patient Factors, Regional Factors, and Crude Odds Ratios for Gastrostomy

### Results of Multilevel Logistic Analyses

Table 3 shows results of the null model and models 1 and 2 after multilevel logistic regression analyses. Females, patients aged ≥90 years, and those with malignancies had significant associations with a lower proportion of gastrostomy. Neurological comorbidities, except for CVA, had significant positive relationships with the proportion of gastrostomy use. CCI and economic status had no relationship with the proportion of gastrostomy, using multivariate analyses. There was significant variance in regional level (MOR 1.71, 1.37–2.49), and MORs between the null model and model 1 were only slightly changed by patient factors (MOR 1.72, 1.37–2.51). In model 2, hospital physician density had a significant relationship with the use of gastrostomy (adjusted OR; 1.75, 1.25–2.45; *P* < 0.001). Moreover, the MOR was 17% lower than for the null model (MOR 1.71 in the null model was reduced to 1.42 in model 2).

**TABLE 3:**
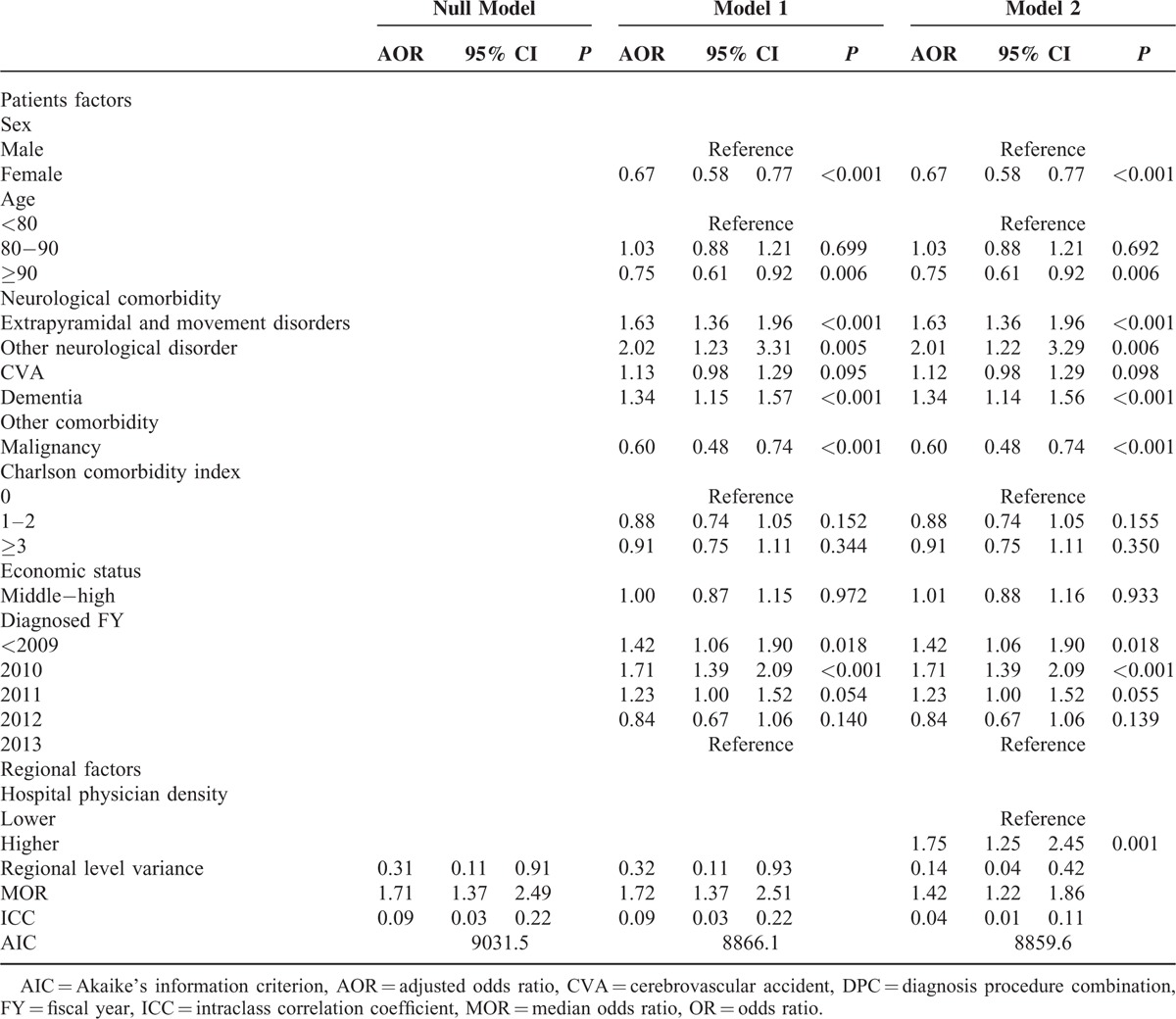
Results of Multilevel Logistic Analyses

### Results of Sensitivity Analyses

In sensitivity analyses (Table 4), the results of model 3 with age tertile in model 2 changed to quartile, those of model 4 with categorized age changed to continuous age, and those of model 5 with economic status tertile in model 2 changed to quartile were all similar to the results of model 2.

**TABLE 4:**
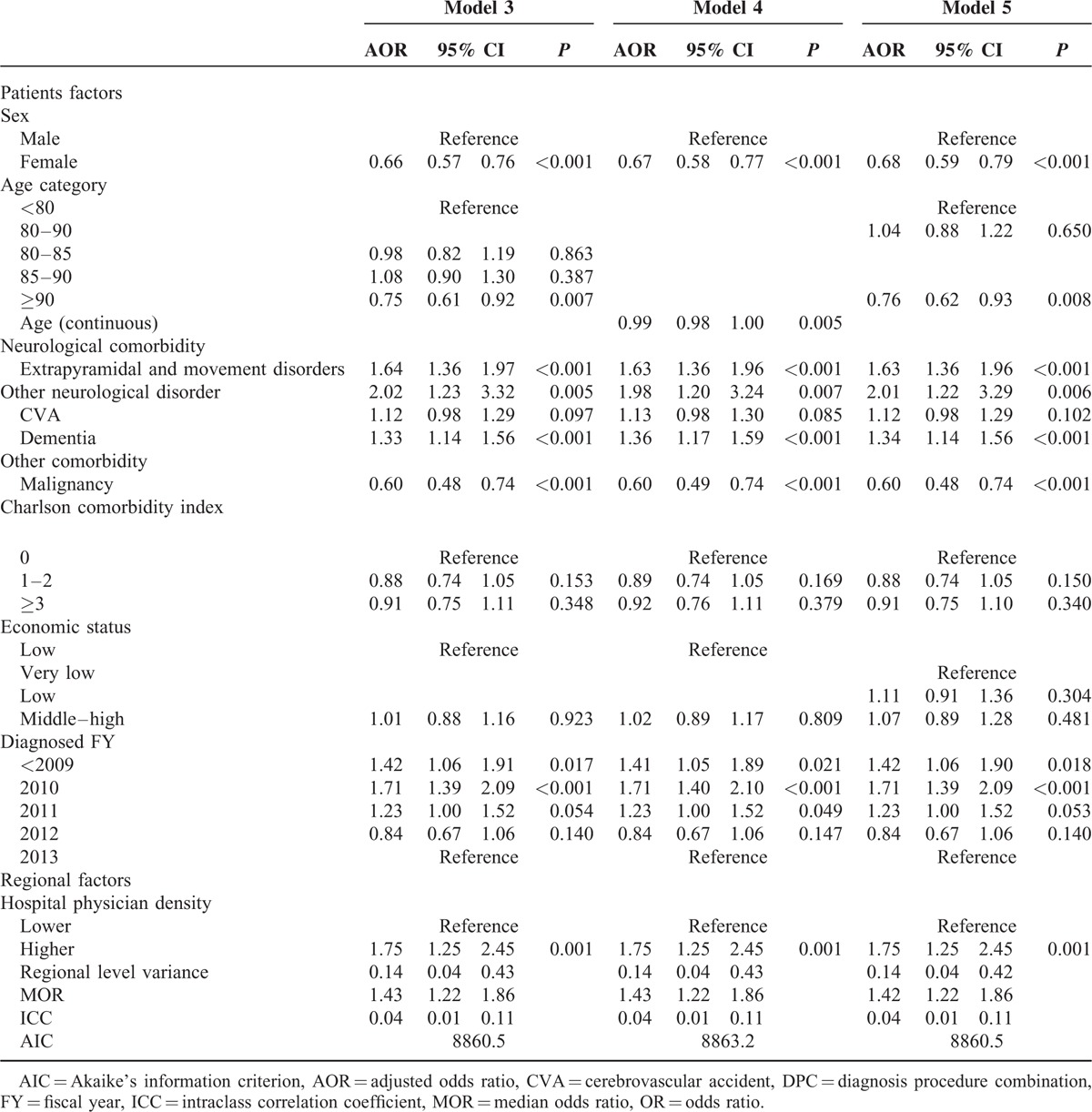
Results of Sensitivity Analyses

## DISCUSSION

Our study aimed to detect the existence of SID for gastrostomy and to investigate the relationships between small area variation in use of gastrostomy and hospital physician density. We found that there was small area variation in gastrostomy use. This could likely be explained by abundant hospital physician density in settings where acute care resources were also abundant, which could in turn indicate the existence of SID. The notion of SID is that doctors, in acting as agents for their patients, may use their “discretionary powers” to shift demand for or encourage certain activities, such that their recommended care differs from care that an informed patient might deem appropriate.^18^ Although SID has been studied for some time, its existence has not been proved because it is difficult to define the appropriate supply of care.^16,21^ However, it has been reported that Japan has a higher proportion of gastrectomy among older adults than other countries,^3,4^ which suggests an oversupply of gastrostomy procedures. Another challenge to detecting SID is that it is difficult to differentiate SID from patient preference.^26^ However, older patients at the end of life do not have the ability to make decisions frequently for themselves. Therefore, family members or caregivers usually decide whether to perform gastrostomy, often at the suggestion of a physician. ^14,17^ It is plausible that SID does exist because it is unlikely that patients themselves demand gastrostomy.

With respect to reasons other than economic ones, gastrostomy might be carried out as an opportunity for resident training in an acute care setting.^27^ Moreover, it has been reported that nursing home residents in Japan had more feeding tubes than in other countries.^4^ This could be because tube feeding is time saving and economically preferable to hand feeding.^28,29^ It is also possible that patients admitted to acute care facilities have gastrostomy tubes placed for transport to other long-term care facilities.^14^

Based on our results, we cannot rule out the possibility that the significant relationship between hospital physician density and gastrostomy is not owing to SID, but rather to physician practice style, uncertainty of clinical outcomes, expectations of family or caregivers, or regional or cultural backgrounds.^16,26,30^ Further studies are needed to clarify whether the variation found here arises from SID or other factors.

It is curious that no relationship was found between patient economic status and gastrostomy. Some studies have reported that small area variations in health care technologies are affected by socioeconomic status.^16,18,31^ Our study was not consistent with those studies, which might be because Late-Elders’ Health Insurance approves all patients for gastrostomy, owing to lower coinsurance rates and reductions in the high cost of health care.

Another outstanding finding is that patients with dementia received more gastrostomy procedures, approximately 30% more than those without dementia. This is despite previous reports that gastrostomy for advanced dementia does not prevent aspiration,^7,8^ pressure sores,^7,10^ or mortality.^7,11,12^ However, our study used claim data; therefore, the stage of patient dementia was unknown.

The strength of this study is that it covered nearly every older adults in Fukuoka Prefecture. A study limitation is that we did not use information about patient daily life activities or laboratory data; instead, we used insurance claim data. In addition, we had no information about advance directives, which have an important role in decision making with respect to gastrostomy.^32^

In conclusion, this study revealed that inappropriate use of gastrostomy in Japan is likely. The use of surveillance systems to monitor quality of care in certain settings is advisable.

## References

[1] White Paper on aging society 2014. Cabinet Off. Gov Japan; 2015(in Japanese)(url;http://www8.cao.go.jp/kourei/whitepaper/w-2014/zenbun/;last access July14)

[2] Clegg A Young J Iliffe S Frailty in elderly people. *Lancet* 2013; 381:752–762. 23395245 10.1016/S0140-6736(12)62167-9 PMC4098658

[3] Sako A Yasunaga H Horiguchi H Prevalence and in-hospital mortality of gastrostomy and jejunostomy in Japan: a retrospective study with a national administrative database. *Gastrointest Endosc* 2014; 80:88–96. 24472760 10.1016/j.gie.2013.12.006

[4] Nakanishi M Hattori K Percutaneous Endoscopic Gastrostomy (PEG) tubes are placed in elderly adults in Japan with advanced dementia regardless of expectation of improvement in quality of life. *J Nutr Heal Aging* 2014; 18:503–509. 10.1007/s12603-014-0011-9 PMC12880495 24886737

[5] Aita K Takahashi M Miyata H Physicians’ attitudes about artificial feeding in older patients with severe cognitive impairment in Japan: a qualitative study. *BMC Geriatr* 2007; 7:22. 17705852 10.1186/1471-2318-7-22 PMC1997114

[6] Report on elderly patients in whom PEG tube is placed and management of tube feeding in residential facilities for elderly and home care settings. All Japan Hosp. Assoc. 2011:1-285.(in Japanese) Available at:(http://www.ajha.or.jp/voice/pdf/other/110416_1.pdf).

[7] Finucane TE Tube feeding in patients with advanced dementia: a review of the evidence. *JAMA* 1999; 282:1365–1370. 10527184 10.1001/jama.282.14.1365

[8] Finucane TE Bynum JP Use of tube feeding to prevent aspiration pneumonia. *Lancet* 1996; 348:1421–1424. 8937283 10.1016/S0140-6736(96)03369-7

[9] Gillick MR Rethinking the role of tube feeding in patients with advanced dementia. *N Engl J Med* 2000; 342:206–210. 10639550 10.1056/NEJM200001203420312

[10] Teno JM Gozalo P Mitchell SL Feeding tubes and the prevention or healing of pressure ulcers. *Arch Intern Med* 2012; 172:697–701. 22782196 10.1001/archinternmed.2012.1200 PMC3555136

[11] Teno JM Gozalo PL Mitchell SL Does feeding tube insertion and its timing improve survival? *J Am Geriatr Soc* 2012; 60:1918–1921. 23002947 10.1111/j.1532-5415.2012.04148.x PMC3470758

[12] Sanders DS Carter MJ D'Silva J Survival analysis in percutaneous endoscopic gastrostomy feeding: a worse outcome in patients with dementia. *Am J Gastroenterol* 2000; 95:1472–1475. 10894581 10.1111/j.1572-0241.2000.02079.x

[13] Guideline on decision-making process in health care for the elderly. Japan Geriatr Soc. 2012:1-24.(in Japanese) Available at: http://www.jpn-geriatsoc.or.jp/info/topics/pdf/jgs_ahn_gl_2012.pdf.

[14] Report of surveillance on gastrostomy and its outcomes. Inst Heal Econ Policy. 2013:1–162.(in Japanses).

[15] Kawakami Y.2012. Japanese fee schedule 2012.37th ed. Tokyo: Reseach for social insurance. (in Japanese).

[16] The Economics of Health and Health Care (7th Edition): Sherman Folland, Allen C. Goodman, Miron Stano.2010 New Jersey : Prentice Hall.

[17] Teno JM Mitchell SL Kuo SK Decision-making and outcomes of feeding tube insertion: a five-state study. *J Am Geriatr Soc* 2011; 59:881–886. 21539524 10.1111/j.1532-5415.2011.03385.x PMC3254052

[18] Ian Bickerdyke, Robert Dolamore, Ian Monday and Robb Preston. Supplier-Induced Demand for Medical Services - Productivity Commission Staff Working Paper. Productivity Commission of Australian Goverment .2002 1–113.

[19] Sundararajan V Henderson T Perry C New ICD-10 version of the Charlson comorbidity index predicted in-hospital mortality. *J Clin Epidemiol* 2004; 57:1288–1294. 15617955 10.1016/j.jclinepi.2004.03.012

[20] Classification for coinsurance rate. Fukuoka late-elders health insurance. (in japanese) url;http://www.fukuoka-kouki.jp/outline05.html Accessed August 11, 2015.

[21] Sekimoto M Ii M Supplier-induced demand for chronic disease care in Japan: multilevel analysis of the association between physician density and physician-patient encounter frequency. *Value Heal Reg Issues* 2015; 6:103–110. 10.1016/j.vhri.2015.03.010 29698180

[22] Larsen K Merlo J Appropriate assessment of neighborhood effects on individual health: integrating random and fixed effects in multilevel logistic regression. *Am J Epidemiol* 2005; 161:81–88. 15615918 10.1093/aje/kwi017

[23] George BP Kelly AG Schneider EB Current practices in feeding tube placement for US acute ischemic stroke inpatients. *Neurology* 2014; 83:874–882. 25098538 10.1212/WNL.0000000000000764 PMC4153849

[24] Cooke CR Kennedy EH Wiitala WL Despite variation in volume, Veterans Affairs hospitals show consistent outcomes among patients with non-postoperative mechanical ventilation∗. *Crit Care Med* 2012; 40:2569–2575. 22732289 10.1097/CCM.0b013e3182591eee

[25] Merlo J Chaix B Ohlsson H A brief conceptual tutorial of multilevel analysis in social epidemiology: using measures of clustering in multilevel logistic regression to investigate contextual phenomena. *J Epidemiol Community Health* 2006; 60:290–297. 16537344 10.1136/jech.2004.029454 PMC2566165

[26] Yasaitis LC Bynum JPW Skinner JS Association between physician supply, local practice norms, and outpatient visit rates. *Med Care* 2013; 51:524–531. 23666491 10.1097/MLR.0b013e3182928f67 PMC3810471

[27] Matsuda S Casemix as a tool for transparency of medical services Shinya Matsuda. *Japanese J Soc Secur Policy* 2007; 6:43–53.

[28] Mitchell SL Buchanan JL Littlehale S Tube-feeding versus hand-feeding nursing home residents with advanced dementia: a cost comparison. *J Am Med Dir Assoc* 2004; 5 (2 suppl):23–29. 10.1097/01.JAM.0000043421.46230.0E 14984607

[29] Mitchell S Financial incentives for placing feeding tubes in nursing home residents with advanced dementia. *J Am Geriatr Soc* 2003; 51:129–131. 12534858

[30] Nattinger AB Gottlieb MS Veum J Geographic variation in the use of breast-conserving treatment for breast cancer. *N Engl J Med* 1992; 326:1102–1107. 1552911 10.1056/NEJM199204233261702

[31] Gould JB1 Davey B Stafford RS.Socioeconomic differences in rates of cesarean section. *N Engl J Med* 1989; 321:233–239. 2747759 10.1056/NEJM198907273210406

[32] Teno JM Mitchell SL Gozalo PL Hospital characteristics associated with feeding tube placement in nursing home residents with advanced cognitive impairment. *JAMA* 2010; 303:544–550. 20145231 10.1001/jama.2010.79 PMC2847277

